# An Alternative Approach to Adult One-Lung Ventilation Using a Standard Single-Lumen Endotracheal Tube

**DOI:** 10.7759/cureus.116437

**Published:** 2026-09-17

**Authors:** Mahesh S Desilva, Dineth L Senanayake

**Affiliations:** 1 Department of Anesthesiology, Riverside Community Hospital, Riverside, USA; 2 Department of Biological Sciences, University of California, Irvine, USA

**Keywords:** endobronchial or mainstem intubation, flexible intubation scope, one-lung ventilation, single-lumen endotracheal tube, video-assisted thoracoscopic surgery

## Abstract

Endobronchial intubation with a double-lumen tube (DLT) is a frequently used method for achieving one-lung ventilation (OLV) in adults, together with simultaneous contralateral lung deflation during intrathoracic surgical procedures. Alternative methods of providing OLV include the bronchial blocker (BB), which comes in several different models (Univent {Columbia, MD: Ambu Inc.}, Arndt blocker {Bloomington, IN: Cook Medical}, Cohen Flex-Tip blocker {Bloomington, IN: Cook Medical}, EZ-Blocker {Morrisville, NC: Teleflex Incorporated}). This case report presents another cost-effective alternative approach to OLV utilizing resources readily found in an operating room setting. This alternative approach to OLV via elective endobronchial intubation of a mainstem bronchus utilizing a standard single-lumen endotracheal tube (ETT) can be especially handy when difficult DLT or BB placements are encountered (difficult airways/severely limited mouth opening/rigid bronchoscopy procedures), in emergent situations involving a unilateral lung (massive bleeding/infection/bronchopleural fistula/pneumothorax), in remote locations out of the operating room or when there are limited resources. It can also be a potential pragmatic way to administer OLV when a patient scheduled for an intrathoracic surgical procedure comes to the operating room already intubated with a single-lumen ETT and will return intubated.

## Introduction

Double-lumen tubes (DLTs) are now considered the gold standard for achieving one-lung ventilation (OLV) and lung isolation during thoracic surgery [1]. They allow for rapid lung collapse, giving surgeons optimal surgical access and visualization, along with the ability to suction blood/secretions from either lung without contaminating or interrupting the ventilation of the other. Left-sided DLT is the most commonly used, while the right-sided DLT incorporates an additional opening slot to permit ventilation of the right upper lobe. At times, DLTs can be difficult to insert due to some of their design characteristics, such as stiffness, larger diameter, and distal curvature, potentially resulting in complications such as hypoxemia, sore throat/hoarseness, and injury to the airways [2].

Prior to the introduction of a functional DLT for thoracic anesthesia by Björk and Carlens in 1950, OLV was achieved by other means [3]. In 1932, Gale and Waters pioneered "closed endobronchial anesthesia" to achieve single-lung isolation by inserting a single, long, cuffed rubber tube (premolded to curve to the left or right) into the trachea using direct laryngoscopy, then blindly advancing it until resistance was met and inflating the cuff [4]. The inflated cuff sealed the intubated bronchus and blocked the carina as well, preventing air from entering the operative side and stopping secretions from spilling into the healthy lung. Since then, single-lumen endobronchial tubes (EBTs) have been improved with longer lengths, smaller external diameters, narrower cuffs, and a shorter distance between the proximal edge of the cuff and the distal tip of the tube. These features made placement in the main bronchi easier and improved the "margin of safety," which is defined as the length of the tracheobronchial tree over which a tube can be moved or positioned without obstructing the conducting airway [5].

In 1936, Magill developed the initial technique of "bronchial blocking," which led to the utilization of bronchial blockers (BB) to help achieve OLV [6]. BBs have since undergone many improvements in design. They are now a common, effective alternative to DLTs to achieve OLV for patients with difficult airways, younger pediatric patients (especially those whose airways are too small for DLTs), patients at high risk of aspiration, patients with tracheostomies, and those patients who already have a single-lumen endotracheal tube (ETT) in place. They can be placed either through the single-lumen ETT or adjacent to it [7,8]. Although BBs typically take longer to place than DLTs, ventilation is quicker to achieve with the initial step of single-lumen ETT placement and hence preferred when some of the above-noted situations are encountered [7].

Other not so commonly used alternatives to achieve OLV, especially for quicker thoracic surgery procedures (such as decortication) or in patients with previous pneumonectomies, include apneic oxygenation. Apneic oxygenation maintains systemic oxygenation without significant lung expansion since mechanical ventilation is paused [9,10]. It relies on the principle of "aventilatory mass flow" or "apneic diffusion of oxygenation," which involves the movement of additional oxygen into the alveoli due to a subatmospheric pressure generated by the diffusion of oxygen from the alveoli into the bloodstream [11]. Because it restricts carbon dioxide (CO_2_) removal, its duration is limited by progressive hypercapnia and respiratory acidosis. While oxygen is maintained, CO_2_ cannot be expelled by passive diffusion. Consequently, arterial partial pressure of CO_2_ (PaCO_2_) rises by about 8-16 mmHg in the first min and roughly 3 mmHg per min thereafter [12]. Despite this restriction, apneic oxygenation can typically provide up to 8-10 min in healthy adults, 3-5 min in obese patients, and 2-3 min or less in critically ill patients, and the duration can be significantly prolonged with the use of high-flow nasal oxygen or oxygen flow in the distal airway via an ETT or tracheal catheter [13].

Nowadays, OLV utilizing an endobronchial (mainstem) intubation with a standard single-lumen ETT is rare, especially in adults. It is still used in young children with smaller-diameter tracheas, and occasionally in adults during unilateral pulmonary emergencies, for quick thoracic procedures or difficult DLT/BB placement airway situations. It is achieved by intentionally advancing a standard single-lumen tube into a mainstem bronchus, preferably with flexible intubation scope (FIS) guidance [14]. This case report re-explores the possibility of elective endobronchial intubation with a standard single-lumen ETT for OLV in adults, similar to its employment prior to the advent of DLTs and BBs. While it is not the first option to achieve OLV in adults, it may offer providers an alternative for quick thoracic procedures such as decortication surgeries, when patients come to the OR already intubated with a single-lumen ETT, when there is a lack of equipment, or when difficult DLT/BB placement airway situations are encountered.

## Case presentation

A 40-year-old male (6 ft 2 in, 100 kg) with a past medical history of paraplegia and a chronic right chest wall wound draining foul-smelling pus was transferred to our institution. The patient was intubated with a 7.5 mm internal diameter (ID) ETT in the emergency room and transferred to the intensive care unit. On hospital day four, a right video-assisted thoracoscopic surgery (VATS) decortication and right chest wall debridement was scheduled. Since the planned surgery was relatively short and potential difficulty with DLT placement was anticipated due to oropharyngeal secretions/swelling, the anesthesia team decided to achieve OLV with a left mainstem intubation using a single-lumen ETT. The 7.5 mm ID ETT was initially removed and replaced with a 6.5 mm ID ETT (Shiley; Minneapolis, MN: Medtronic) using GlideScope Core video laryngoscope guidance (Bothell, WA: Verathon Inc.) because the latter ETT has a smaller cuff that could be accommodated within the left mainstem bronchus. Also, since the 6.5 mm single-lumen ETT was anticipated to recede farther into the mouth during mainstem intubation, it was first extended by connecting it to another proximal portion of a 6.5 mm ID ETT via a cut 6.0 mm male connector (Medi-Vac; Ontario, CA: Cardinal Health) found in 6.0 mm surgical suction tubing kits (Figures 1, 2).

**Figure 1:**
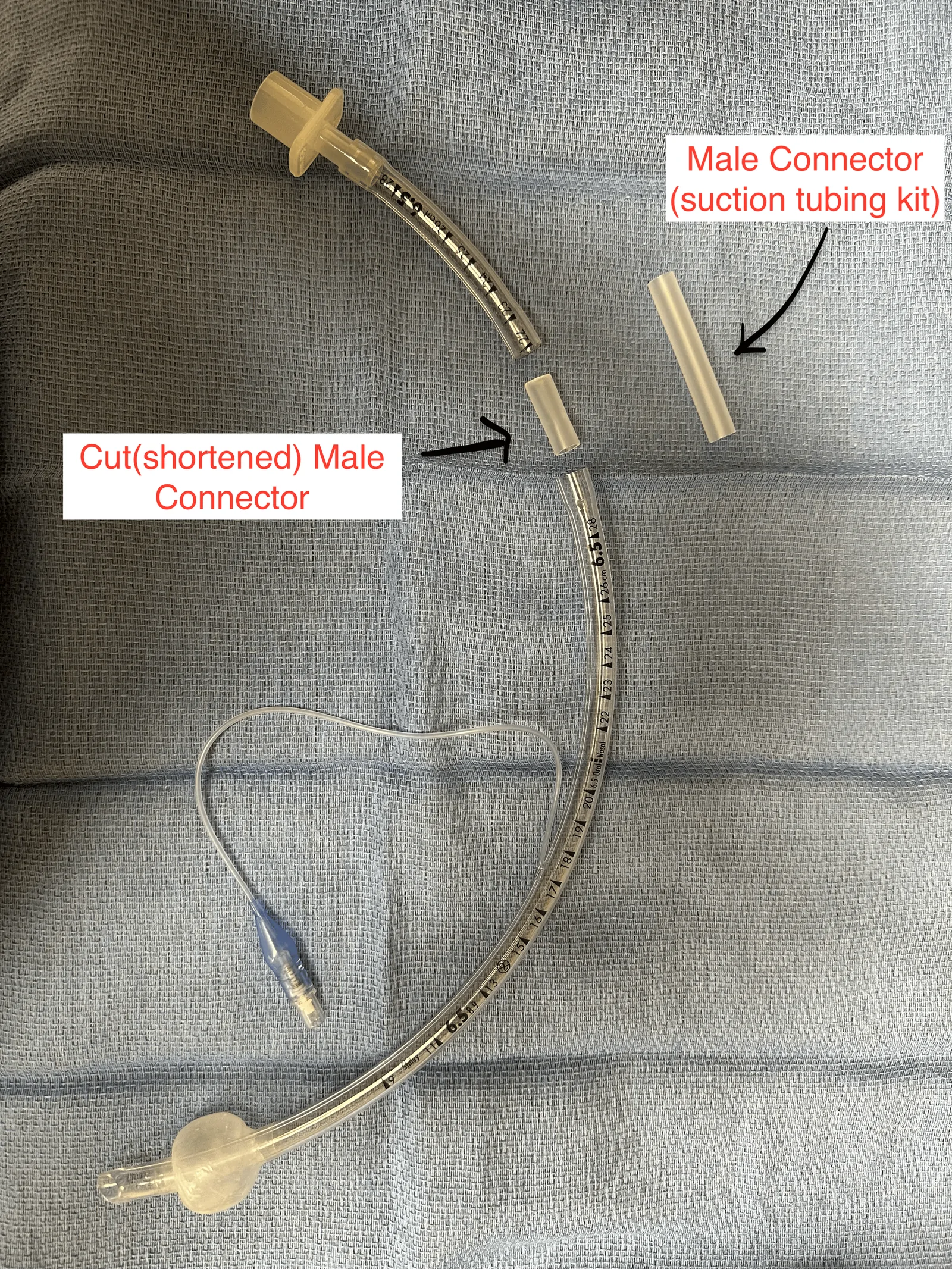
Extension of a single-lumen ETT using a cut male connector (from suction tubing kit). ETT: endotracheal tube

**Figure 2:**
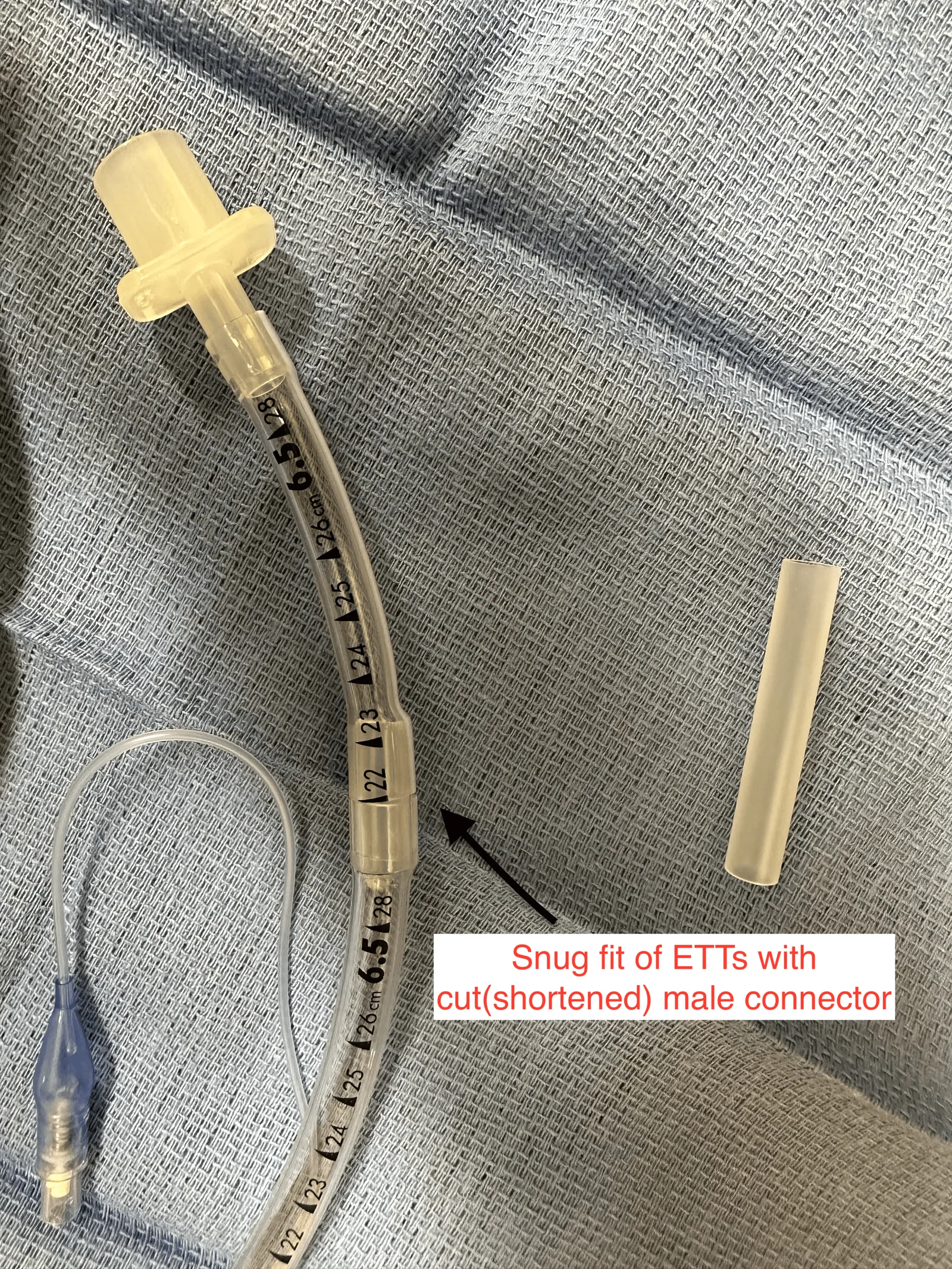
Snug fit of the proximal and distal 6.5 single-lumen ETTs connected by a cut male connector (from suction tubing kit). ETT: endotracheal tube

A 5.5 mm × 65 cm flexible intubation scope (FIS) (Tuttlingen, Germany: KARL STORZ SE & Co. KG) was then inserted through the extended 6.5 mm ID ETT to guide its entry into the left main bronchus, and the ETT was positioned just before the bifurcation of the left main bronchus into the lobar (secondary) bronchi (Figure 3, Video 1).

**Figure 3:**
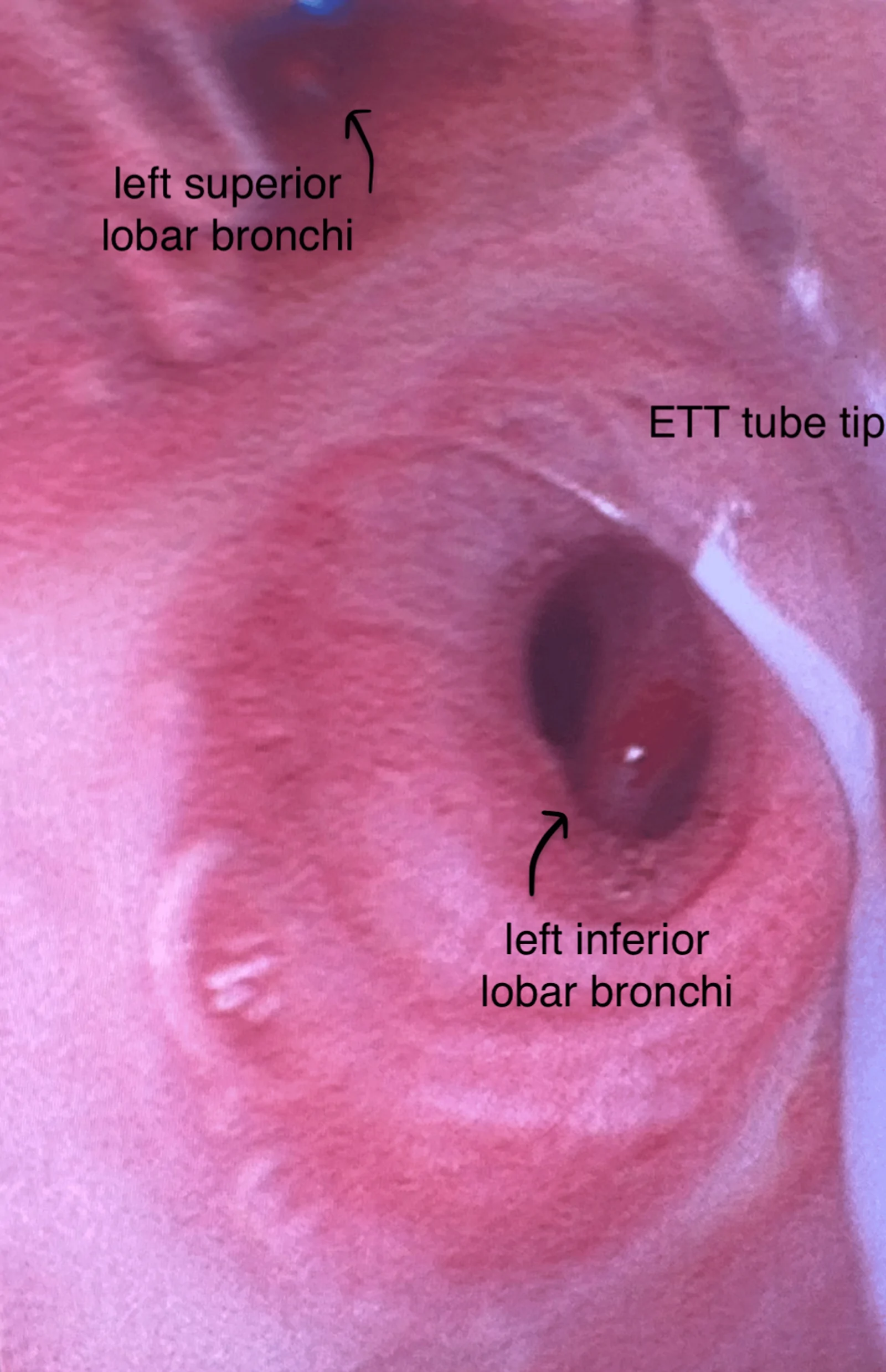
FIS-guided placement of the single-lumen ETT tip in the left main bronchus just before its division into the left superior and inferior lobar bronchi. FIS: flexible intubation scope; ETT: endotracheal tube

**Video 1:** Clinical video demonstrating FIS-guided adjustment of the single-lumen ETT in the left mainstem bronchus before its division into the left superior and inferior lobar bronchi to ensure unobstructed one-lung ventilation. FIS: flexible intubation scope; ETT: endotracheal tube; OLV: one-lung ventilation

After OLV was initiated in the left lung, minimal ETT cuff herniation into the trachea was confirmed by advancing a FIS through the glottis adjacent to the 6.5 mm ETT and visualizing at the carina (Figure 4). Adequate right lung isolation was confirmed after trocar placement to proceed with the right VATS decortication (Figure 5).

**Figure 4:**
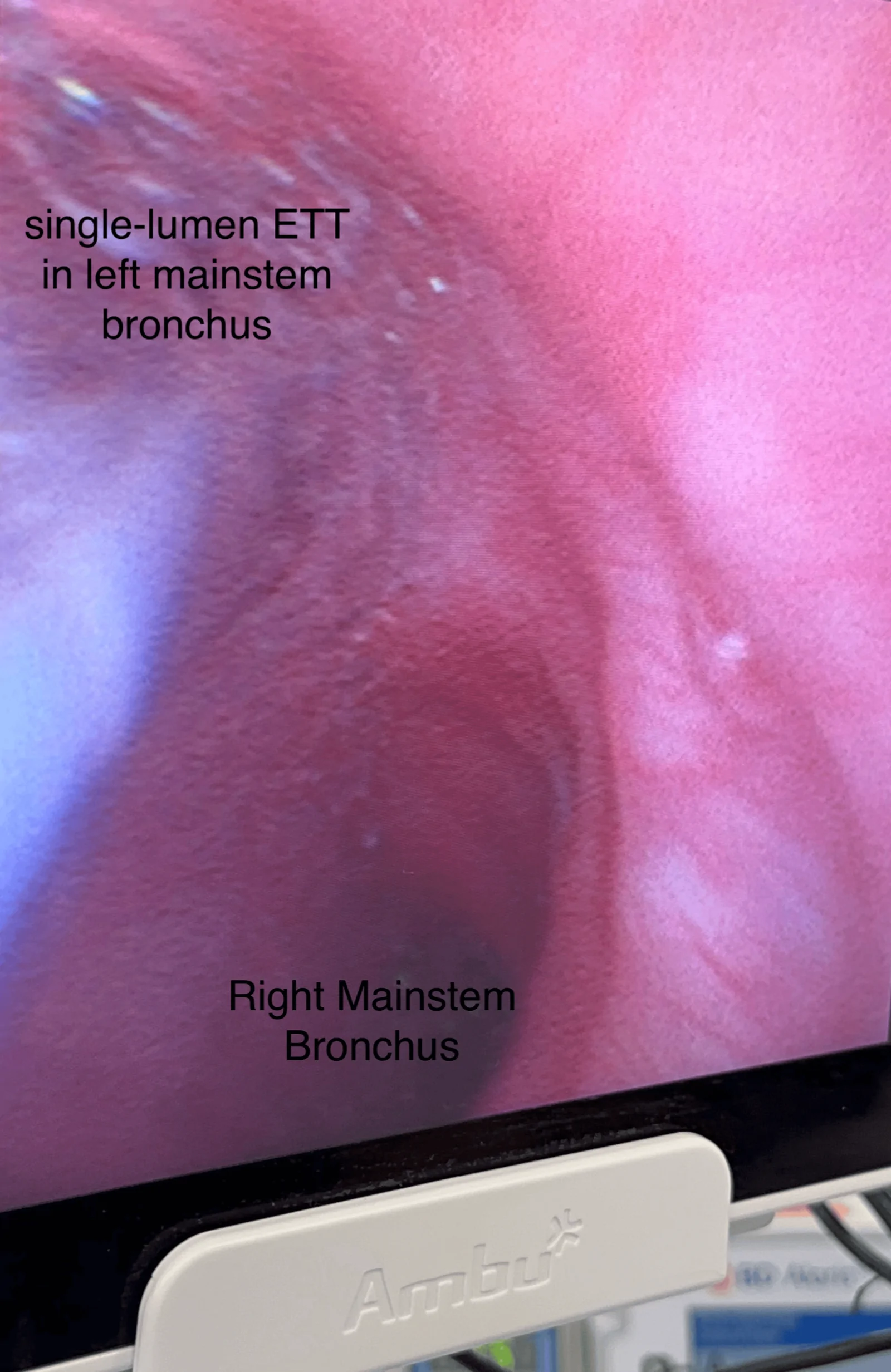
FIS carinal view with the single-lumen ETT situated in the left main bronchus (without herniation of its cuff into the trachea). FIS: flexible intubation scope; ETT: endotracheal tube

**Figure 5:**
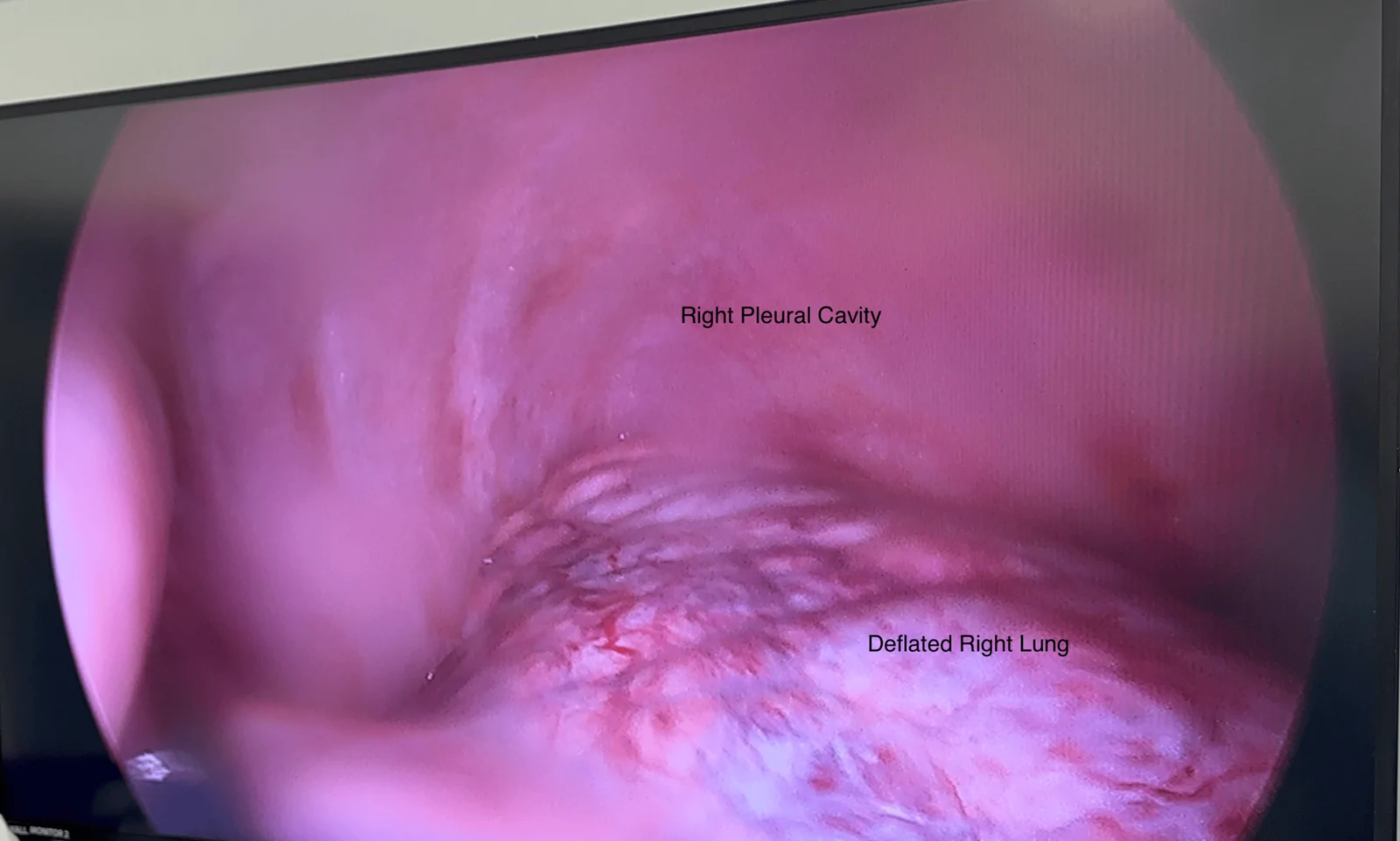
Adequate deflation of the right lung and thoracic cavity exposure after initiation of OLV via left endobronchial single-lumen ETT intubation. OLV: one-lung ventilation; ETT: endotracheal tube

No anesthesia circuit leak was noted during the surgery, and the patient tolerated the procedure with stable hemodynamics, oxygenation, and ventilation (Figure 6). The patient was extubated at the end of the procedure, and no anesthesia-related complications were noted perioperatively. An animated video of the steps used to achieve OLV via a standard single-lumen ETT in the left main bronchus, as shown in this case presentation, is attached below to help visualize the technique (Video 2).

**Figure 6:**
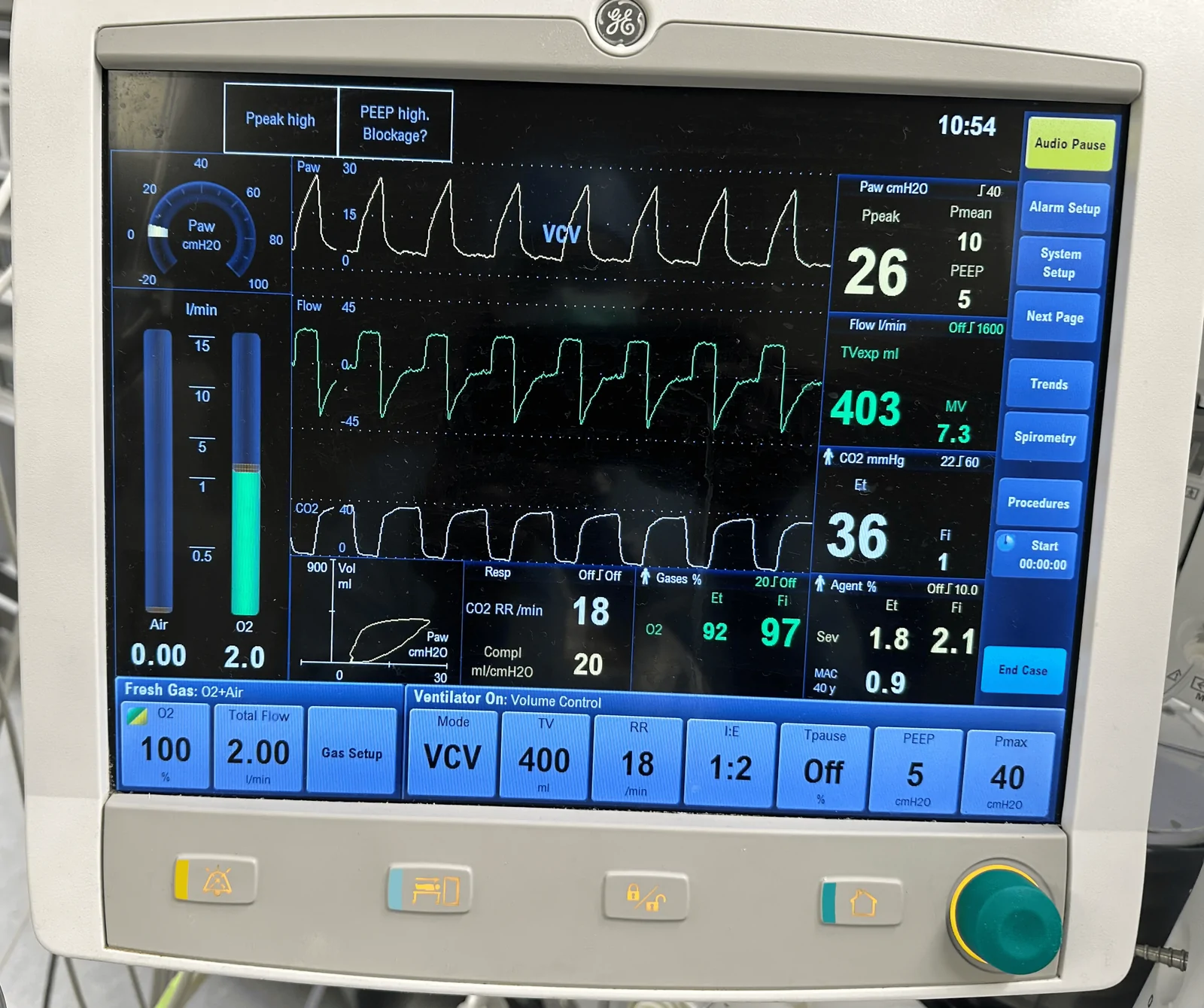
Intraoperative monitoring showing attainment of set tidal volume in the left lung (signifying no significant leak) and acceptable peak pressures after initiation of OLV via a left endobronchial single-lumen ETT. OLV: one-lung ventilation; ETT: endotracheal tube; PEEP: positive end-expiratory pressure

**Video 2:** This is an animated video of OLV utilizing elective intubation of the left mainstem bronchus with a single-lumen ETT. The patient is first intubated with a single-lumen ETT, ideally with a 6.5-7.0 mm ID. A FIS is then used to guide the single-lumen ETT tip into the left mainstem bronchus and position it immediately proximal to its bifurcation into the left superior and inferior lobar bronchi. Next, the FIS is removed, and OLV is initiated in the left lung with deflation of the right lung. FIS is then passed again adjacent to the ETT at the glottis and advanced until a carinal view is obtained to confirm minimal herniation of the ETT cuff into the trachea. Graphic artist Phoenix Rose Hoffman developed this animated video illustrating the technique under the guidance of Dr. Mahesh Desilva, and permission has been granted to use the video for publication. OLV: one-lung ventilation; ETT: endotracheal tube; FIS: flexible intubation scope; ID: internal diameter

## Discussion

OLV is an essential requirement for performing many intrathoracic surgical procedures, such as stapling of bullae, pleurodesis, lung biopsy/resection, and thoracic sympathectomy safely and efficiently. The deflation of the lung on the surgical side provides room to operate with surgical tools in the chest cavity. Anesthesia providers can choose among different airway management options to obtain adequate lung isolation. The most commonly used approaches to OLV in adults are via placement of a DLT or a BB through a single-lumen ETT [15-17].

A single-lumen ETT alone can also be used to achieve OLV in adults via selective intubation of a mainstem bronchus. As noted in the introduction, this approach was often utilized prior to the advent of BBs and DLTs. More recently, its use has been documented in emergent situations such as unilateral massive pulmonary hemorrhage from a bronchial tumor or with rigid bronchoscopy procedures [18,19]. Elective endobronchial intubations with single-lumen ETTs are also performed in pediatric anesthesia in lieu of a DLT, which is too large to fit the airways of smaller pediatric patients [20-22].

One of the modern single-lumen endobronchial tubes (EBT) engineered specifically for OLV is the Fuji wire-reinforced endotracheal tube (WRETT) (Columbia, MD: Ambu Inc.) [23,24]. The WRETT tube is wire-reinforced, longer (40 cm), has a shorter non-beveled tip without a Murphy eye, and a shorter cuff, which can seal more easily in the mainstem bronchi (Figure 7).

**Figure 7:**
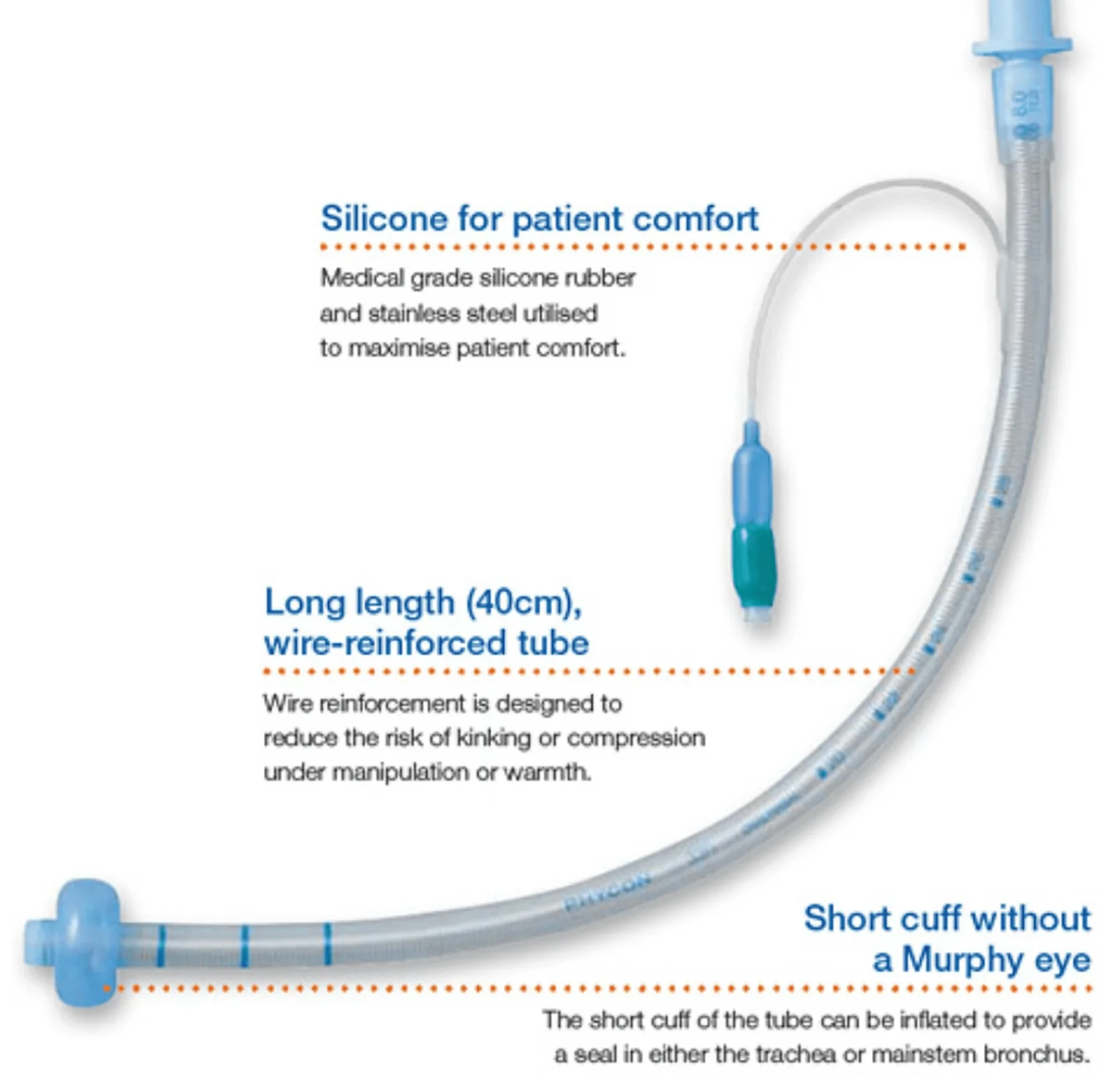
Commercially available Fuji WRETT (Columbia, MD: Ambu Inc.) tube. Uniquely constructed single-lumen ETT with longer length, wire-reinforcement, and a short cuff to allow for easier endobronchial placement to enable OLV. WRETT: wire-reinforced endotracheal tube; ETT: endotracheal tube; OLV: one-lung ventilation

In this study, the authors electively used an extended standard single-lumen 6.5 mm ETT to achieve adequate OLV for a right lung video-assisted thoracoscopic surgery (VATS) decortication in an adult. Continuous FIS visualization at the carina to confirm the absence of endobronchial ETT cuff herniation into the trachea was crucial for executing the anesthetic plan.

The left main bronchus in adults, before dividing into the superior and inferior lobar bronchi, is especially amenable to selective endobronchial intubation since it is about 5 cm long [25]. This usually allows adequate length to place a single-lumen ETT cuff without a significant herniation/leak into the trachea. The average diameter of the left mainstem bronchus in adults is approximately 10-13 mm [26]. Smaller-sized ETTs have a smaller cuff, so a better fit is more likely with a 6.5 mm ID ETT cuff as opposed to a 7.5 mm ID ETT cuff. Hence, in this study, a 6.5 mm ID ETT was chosen to secure OLV with a smaller-sized ETT cuff after removing the 7.5 mm ID ETT in situ. The distal tip of the beveled orifice of a 6.5 mm ID ETT to the proximal cuff (cuff-tip distance) is 4.5-6 cm depending on the manufacturer [27]. The cuff of an ETT can also be reduced in size using suture thread and Tegaderm to improve the likelihood of an endobronchial fit, as shown by Schultz et al. (Figure 8) [18].

**Figure 8:**
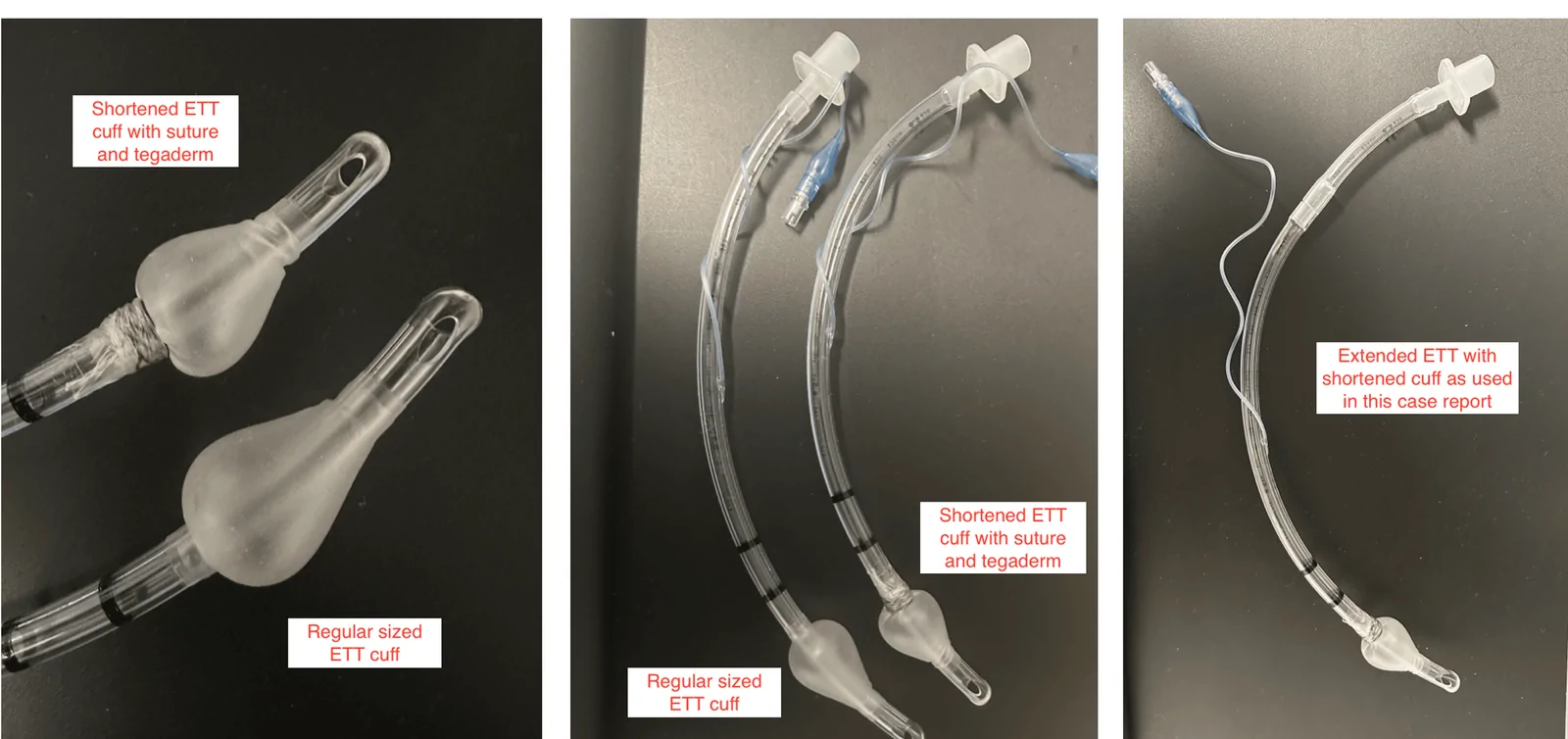
Shortening of a single-lumen ETT cuff using sutures and Tegaderm tape to reduce the chance of cuff herniation into the trachea with selective endobronchial intubation, especially on the right side. ETT: endotracheal tube

This ETT modification, along with elongation of the Murphy eye, is especially useful in selective endobronchial intubation of the shorter right mainstem bronchus, which only averages about 2.5 cm in length before takeoff of the right upper lobe bronchus [28]. This technique also allows larger-sized ETTs (7.0-7.5 mm ID) to be potentially used to secure one-lung ventilation, which would otherwise only be possible with smaller-sized ETTs (6.0-6.5 mm ID). Furthermore, for right mainstem endobronchial intubation, the ETT will have to be placed with FIS guidance, and the tip rotated and maneuvered to avoid obstructing the right upper lobe bronchus. A potential problem with using smaller-sized ETTs or shortened cuffs is that they may not adequately dilate to fit the trachea to resume two-lung ventilation at the end of surgery, potentially requiring a swap with a properly sized ETT and cuff adequate for tracheal ventilation.

Visualization at the carina with a FIS is an excellent way to monitor and troubleshoot inadequate OLV with a single-lumen ETT. It can assist the anesthesia provider in adjusting and maintaining optimal endobronchial position of the ETT cuff. The authors achieved this visualization continuously (similar to an Ambu VivaSight 2 DLT; Columbia, MD: Ambu Inc.) via advancement and placement of the FIS tube adjacent to the single-lumen ETT in the trachea. This is similar to how McLaurin et al. passed a bronchial blocker adjacent to a single-lumen ETT to facilitate tracheobronchoplasty repair [29]. If secretions are noted at the carinal view, then suction/flushes can be applied with FIS to potentially improve the view.

Extension of the single-lumen ETT is an optional step that can help situate the proximal end of the ETT in a more ideal, easy-to-reach location outside the mouth, as opposed to having it sunken inside the mouth. Several techniques have been employed to extend an ETT, including the extra-long single-lumen “super tube” fashioned by McLaurin et al. to facilitate a tracheobronchoplasty [18,29,30]. In this study, the connection/extension of the 6.5 mm single-lumen ETT was uniquely and successfully achieved using the readily available 6.0 mm male connector from a 6.0 mm Medi-Vac surgical suction tubing kit, which is much easier to cut and shorten than the ETT connectors used by others. Such extension of an endotracheal tube with a smaller-diameter connector does increase dead space, resistance, and turbulence and can possibly trap passively exhaled expired air from the elastic recoil of the lungs. This can potentially lead to hypercapnia, reduced alveolar ventilation, and gas trapping (auto-positive end-expiratory pressure {PEEP}). However, studies have shown no increased risk of barotrauma and clinically acceptable positive end-expiratory pressures with smaller ETTs of 6.0 mm or larger [31,32]. Yet if the single-lumen ETT is extended, close attention must be paid to ensure adequate tidal volume settings, acceptable EtCO2 levels, and airway pressures (Ppeak and Pplateau), along with appropriate return to zero flow before the next inspiration on the flow waveform [33]. Furthermore, this extension should preferably be removed prior to resumption of spontaneous ventilation to avoid extra work of breathing for the patient upon emergence.

Limitations associated with using a single-lumen ETT for endobronchial intubation include lobar bronchi obstruction or airway leak with inadequate contralateral lung deflation [34]. Suctioning the non-ventilated lung is difficult, and the technique is incapable of applying selective continuous positive airway pressure (CPAP) to the operative lung.

## Conclusions

A standard single-lumen ETT can be used to achieve adequate OLV in adults via selective endobronchial intubation, as demonstrated in this study. Furthermore, the technique can be facilitated by the extension of the single-lumen ETT, shortening of the single-lumen ETT cuff, and continuous FIS visualization at the carina adjacent to the single-lumen ETT. Although its clinical utility is limited to certain situations, more familiarity with the technique will allow clinicians to apply this technique for achieving OLV.
